# Oligodendrocyte precursor cells sculpt the visual system by regulating axonal remodeling

**DOI:** 10.1038/s41593-022-01023-7

**Published:** 2022-03-03

**Authors:** Yan Xiao, Luigi Petrucco, Laura J. Hoodless, Ruben Portugues, Tim Czopka

**Affiliations:** 1 Institute of Neuronal Cell Biology, Technical University of Munich, Munich, Germany; 2 Max Planck Institute of Neurobiology, Sensorimotor Control Research Group, Martinsried, Germany; 3 Institute of Neuroscience, Technical University of Munich, Munich, Germany; 4 Centre for Clinical Brain Sciences, University of Edinburgh, Edinburgh, UK; 5 Munich Cluster for Systems Neurology (SyNergy), Munich, Germany

**Keywords:** Oligodendrocyte, Axon and dendritic guidance

## Abstract

Many oligodendrocyte precursor cells (OPCs) do not differentiate to form myelin, suggesting additional roles of this cell population. The zebrafish optic tectum contains OPCs in regions devoid of myelin. Elimination of these OPCs impaired precise control of retinal ganglion cell axon arbor size during formation and maturation of retinotectal connectivity and degraded functional processing of visual stimuli. Therefore, OPCs fine-tune neural circuits independently of their canonical role to make myelin.

## Abstract (web summary)

Oligodendrocyte precursor cells exist in abundance throughout the brain lifelong, with unclear functions. Xiao et al. show that, in zebrafish, these cells regulate the precise formation of retinal ganglion cell arbors and fine-tune visual processing.

## Main

Oligodendrocytes play crucial roles in modulating information processing through regulation of axon conduction and metabolism^1,2^. Myelinating oligodendrocytes arise by differentiation of OPCs, which are uniformly distributed across the central nervous system (CNS) and tile the tissue with their elaborate process networks^3^. The formation of new myelin through oligodendrocyte differentiation continues into adulthood and can dynamically change to shape axonal myelination^4–6^. However, the CNS comprises more OPCs than ever differentiate, making about 5% of all CNS cells lifelong^7^. How this persistent population of resident CNS cells affects the CNS apart from being the cellular source of new myelin is largely unclear.

OPCs are a heterogenous population with different properties^8,9^. Clonal analyses have shown that a large proportion of OPCs do not directly generate myelinating oligodendrocytes, suggesting that these cells might have additional physiological functions in the healthy CNS^10^. Indeed, OPCs express molecules that can affect form and function of neurons^11–13^, and altered gene expression in OPCs has recently been linked to mood disorders in humans. However, as changes in OPCs also affect myelination, it remains unclear if roles in the formation of a functional neural circuit can be directly attributed to OPCs that are independent of myelination.

**Fig. 1:**
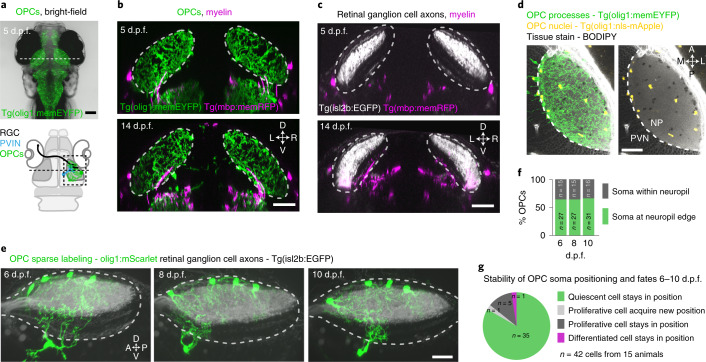
The tectal neuropil of larval zebrafish is interspersed with OPC processes but largely devoid of myelin. **a**, Transgenic zebrafish showing OPC processes throughout the brain. Dashed line indicates cross-sectional plane shown in **b** and **c**. Schematic of zebrafish brain delineates RGC axons, dendrites of PVINs and OPCs in tectal neuropil. **b**, **c**, Cross-sectional views of transgenic zebrafish showing that OPC processes intersperse the tectal neuropil (dashed lines), whereas myelin is largely absent. Scale bars, 50 µm. **d**, Sub-projection of OPC reporter lines (dorsal view) stained with BODIPY to outline tectal neuropil (NP) and the periventricular neuron zone (PVN). Dashed lines indicate the border between NP and PVN. Scale bar, 40 µm. **e**, Time lapse of four individual OPCs (lateral rotation view). Dashed lines depict tectal NP. Scale bar, 20 µm. **f**, **g**, Quantifications of individual OPCs as shown in **e**, showing low rates of soma position changes, division and differentiation.

**Extended Data Fig. 1:**
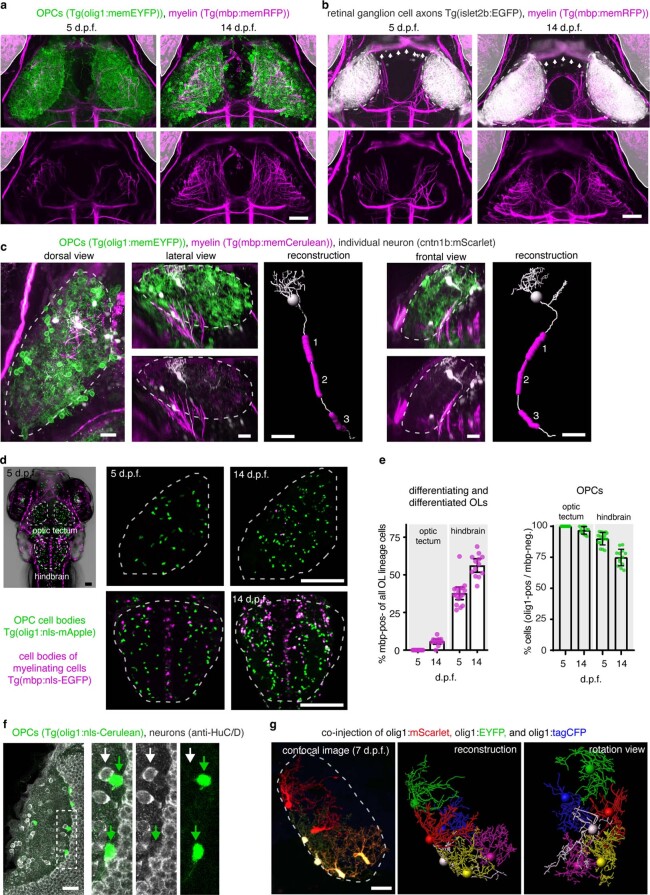
Characterization of OPCs and myelin in the zebrafish tectum. **a** and **b)** Confocal images showing dorsal views of the zebrafish visual system at 5 and 14 dpf in transgenic reporter lines for OPCs, myelin, and RGC axons as indicated. Grayed areas indicate position of the eyes. Arrows in (b) point to myelinated optic nerves. Dashed lines indicate RGC axons in tectal neuropil. Representative images from 8 animals in two independent experiments (a), 9 animals in two independent experiments (b). Scale bars: 50 µm. **c)** Confocal images and 3D reconstructions of individual neuron soma residing within the tectal neuropil and extending intermittently myelinated axon out of the tectal neuropil into deeper brain regions at 9 dpf. Dashed lines outline the tectal neuropil. Representative images from 4 animals in three independent experiments. Scale bars: 20 µm. **d)** Transgenic reporter animals showing mOLs and OPCs in the optic tectum and hindbrain. Left: dorsal view of Tg(mbp:nls-EGFP), Tg(olig1:nls-mApple) at 5 d.p.f. showing mOLs and OPCs, respectively. Dashed lines indicate optic tectum and hindbrain regions. Middle and right panels: Clipped subprojections showing OPCs and mOLs that specifically reside within optic tectum (top) and hindbrain (bottom) at 5 d.p.f (middle) and 14 d.p.f (right), respectively. n values are given in (e). Scale bars: 50 µm. **e)** Left: differentiating and differentiated mOLs expressed as ratio between mbp:nls-EGFP-positive and total OL lineage cells including OPCs (olig1:nls-mApple-pos; mbp:nls-EGFP-neg) and mOLs (mbp:nls-EGFP-pos).Data are expressed as mean ± S.D. (0.0 ± 0.0% in tectal *vs*. 37.7 ± 8.1% in hindbrain at 5 d.p.f.; 5.7 ± 2.5% in tectal *vs*. 56.2 ± 7.4% in hindbrain at 14 d.p.f. Right: undifferentiated OPCs expressed as ratio between OPCs (olig1:nls-mApple-pos; mbp:nls-EGFP-neg) and all the olig1:nls-mApple-pos cells in the optic tectum and hindbrain. (mean 100 ± 0% S.D. in tectal *vs*. 90.0 ± 5.0% in hindbrain at 5 d.p.f.; 96.7 ± 3.0% in tectal *vs*. 74.8 ± 6.7% in hindbrain at 14 d.p.f). n=14/12 at 5/14 dpf in tectum, and n=17/13 at 5/14 d.p.f.in hindbrain from two experiments in both left and right quantitative figures). **f)** Whole mount immunohistochemistry showing that OPCs labeled in transgenic olig1 reporter lines (green arrows) are negative for the pan-neuronal maker HuC/D (white arrows). Representative images from 20 animals in six independent experiments. Scale bar: 20 µm. **g)** Multicolor labeling and 3D reconstruction of OPCs in the tectal neuropil showing that processes of individual cells occupy non-overlapping territories at 7 dpf. Representative images from 23 animals in eight independent experiments. Dashed lines outline the tectal neuropil. Scale bar: 20 µm.

To reveal myelination-independent roles for OPCs, we identified the optic tectum of larval zebrafish as a brain area that is densely interspersed with OPCs that rarely differentiate to oligodendrocytes (Fig. 1, Extended Data Fig. 1a–c and Supplementary Videos 1 and 2). Transgenic lines labeling retinal ganglion cell (RGC) axons as primary input to the tectum (Tg(isl2b:EGFP)), OPCs (Tg(olig1:memEYFP)) and myelin (Tg(mbp:memRFP)) allowed the carrying out of high-resolution, whole-brain imaging of neuron–oligodendrocyte interactions. Myelination of RGC axons was observed along the optic nerve and along tectal neuron axons projecting to deep brain areas (Extended Data Fig. 1a–c). However, the tectal neuropil where RGC axons connect to tectal neuron dendrites remained largely devoid of myelin until at least 14 days post-fertilization (d.p.f.) despite being interspersed with OPC processes throughout (Fig. 1b,c). Quantification of oligodendrocyte numbers confirmed that no more than 6% of oligodendrocyte lineage cells differentiated by 14 d.p.f., in strong contrast to hindbrain regions showing 56% differentiation (Extended Data Fig. 1d,e). Within the tectum, OPCs localized their soma either at the border between the neuropil and periventricular zone containing most tectal neurons or right within the neuropil (Fig. 1d–f and Extended Data Fig. 1f) and claimed non-overlapping territories, similarly to previous studies (Extended Data Fig. 1g and Supplementary Video 3)^10,14,15^. OPCs can be highly dynamic and potentially migrate, proliferate or differentiate. However, sparse labeling revealed that soma positioning of individual tectal OPCs remained largely stable, with a low rate of division and differentiation between 6 d.p.f. and 10 d.p.f. (Fig. 1f,g).

**Fig. 2:**
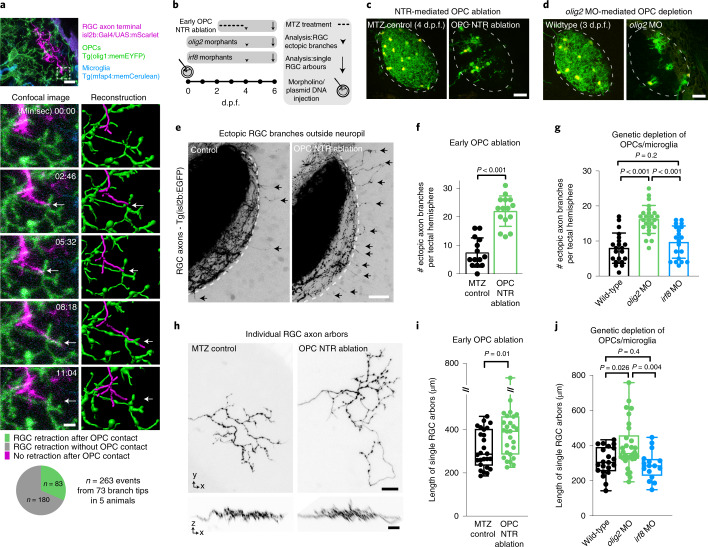
Early OPC depletion causes formation of aberrant RGC arborizations. **a**, Time lapse showing dynamic interactions between RGC axon arbors and OPC processes. Dashed box indicates the position of time lapse shown. Arrows indicate when extending RGC process interdigitates with OPC process and subsequently retracts (see Supplementary Video 6 for 3D rotation to demonstrate contact). Pie chart shows frequency of RGC retractions with and without prior OPC contact. Scale bars, 10 µm (top) and 2 µm (bottom). **b**, Timelines of manipulations in this figure. **c**, **d**, Example images of NTR-mediated OPC ablation (**c**) and olig2 morpholino-mediated OPC depletion. Dashed lines indicate neuropil. Scale bars, 25 µm. **e**, **f**, Increased formation of ectopic RGC axon branches extending outside tectal neuropil (arrows) upon early ablation of OPCs (mean 7.5 ± 5.1 s.d. in control versus 22.0 ± 5.3 in OPC NTR ablation, *n* = 14/15 animals from four experiments, unpaired two-tailed *t*-test, *t* = 7.466, d.f. = 27). Dashed lines indicate the border between NP and PVN. Scale bar, 20 µm. **g**, Increased formation of ectopic RGC axon branches upon genetic OPC reduction (*olig2* morphants) but not upon microglial depletion (*irf8* morphants) (mean 7.9 ± 4.3 s.d. in control versus 16.2 ± 3.9 in *olig2* MO versus 9.7 ± 4.6 in *irf8* MO, *n* = 21/25/20 animals from three experiments, one-way ANOVA, *F*_2,63_ = 23.69). **h**, **i**, Increased size of single RGC arbors upon early OPC ablation (top) while maintaining single lamina layering (bottom) (median 287 ± 403/235 IQR in control versus 393 ± 461/286 in OPC NTR ablation, *n* = 26/27 axons in 24/23 animals from three experiments, two-tailed Mann–Whitney *U*-test, *U* = 210). Scale bars, 10 µm. **j**, Increased size of single RGC arbors upon genetic OPC depletion (*olig2* morphants) but not upon microglial depletion (*irf8* morphants) (median 304 ± 391/255 IQR in control versus 355 ± 458/326 in *olig2* MO versus 285 ± 323/229 in *irf8* MO, *n* = 21/30/16 axons in 11/14/12 animals from five experiments, Kruskal–Wallis test, test statistic = 9.9). MO, morphant.

**Extended Data Fig. 2:**
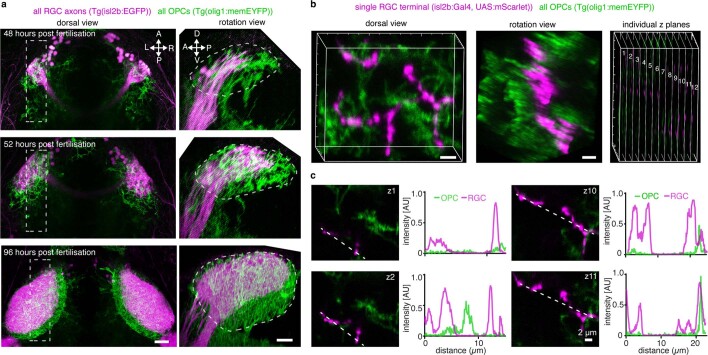
Presence of OPCs during tectal development. **a)** Dorsal (left) and rotation (right) views of confocal images showing the presence of OPCs from 48 hours post fertilization when RGC axons arrive in the tectum. Dashed rectangles indicate area shown in rotation views. Representative images from 4 animals in two independent experiments. Dashed lines indicate tectal neuropil. Scale bars: 20 µm. **b)** Dorsal view (left), rotation view (middle) and individual z planes (right) of confocal images showing single RGC axon interspersed with OPC processes. Representative images from 12 animals in four independent experiments. Scale bars: 2 µm. **c)** Example z planes and fluorescence intensity plots along dashed lines showing RGC axons occasionally contacting OPC processes but largely devoid of OPC processes (n numbers as in (b)).

**Extended Data Fig. 3:**
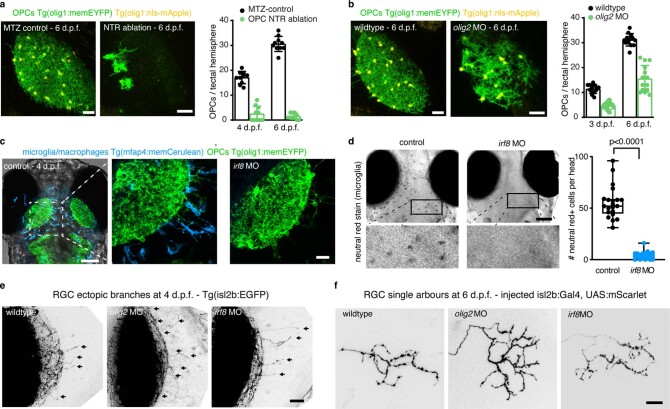
Early ablation and depletion of OPCs and microglia. **a)** Example images of OPCs in tectal hemisphere and quantifications revealing efficient ablation from in olig1:CFP-NTR animals upon MTZ application from 2 d.p.f. Data are expressed as mean ± S.D. (17.1 ± 2.5 in MTZ-control *vs*. 2.5 ± 3.0 in OPC NTR ablation at 4 d.p.f.; 30.6 ± 3.0 in MTZ-control *vs*. 1.5 ± 1.2 in OPC NTR ablation at 6 d.p.f.; n=10 animals per group from two experiments). Scale bar: 25 µm. **b)** Example images of OPCs in tectal hemisphere and quantifications revealing genetic depletion of OPCs in *olig2* morphants. Data are expressed as mean ± S.D. (11.4 ± 1.8 in wildtype *vs*. 4.7 ± 1.4 in *olig2* morphants at 3 d.p.f.; 31.2 ± 2.5 in wildtype *vs*. 15.5 ± 5.3 in *olig2* morphants at 6 d.p.f.; n=13/14 tectal hemisphere in 13/14 wildtype/*olig2* MO from three experiments). Scale bar: 25 µm. **c)** Dorsal views of transgenic reporter lines showing presence of OPC within the tectal neuropil while microglia largely reside outside the neuropil. Dashed box indicates area shown in middle panel. Scale bar: 100 µm. Right panel showing the same transgenic reporter line injected with a morpholino against *irf8* revealing the absence of microglia whilst OPCs remain in position. Representative images from 10 animals in three independent experiments both in control and in *ifr8* MO. Scale bar: 20 µm. **d)** Brightfield images of neutral red stained control and *irf8* morphant animals showing the absence of phagocytic cells at 4 d.p.f. Boxes represent the median (center line) and I.Q.R. (52 ± 45/58.5 cells in control *vs* 3.5 ± 0.75/6 cells in *irf8* morphant animals), and whiskers indicate the minimum and maximum values. (n=21/22 animals in control/*irf8* MO from six experiments, two-tailed Mann-Whitney U test, U=0). Scale bar: 100 µm. **e)** Confocal images showing RGC axons in tectal neuropil. Arrows indicate ectopic branches extending outside the tectal neuropil in wildtypes, *olig2* and *irf8* morphants, respectively. n values are given in Fig. 2g. Scale bar: 20 µm. **f)** Example images of single RGC axon arbors in wildtypes, *olig2* morphants and *irf8* morphants. n values are given in Fig. 2j. Scale bar: 10 µm.

The appearance of OPCs coincided with the time when RGC axons arrive in the developing tectum and establish their terminal arborizations (Extended Data Fig. 2a). Time-lapse imaging revealed that RGC arbors dynamically interact with OPC processes and that interactions frequently preceded retractions of arbor tips (Fig. 2a, Extended Data Fig. 2b,c and Supplementary Videos 4–7). This correlation prompted us to ask whether OPCs influence the organization of RGC arbors. We tested this using an inducible nitroreductase (NTR)-mediated cell ablation system specifically targeted to OPCs (Tg(olig1:CFP-NTR)) (Fig. 2b,c and Extended Data Fig. 3a). Early OPC ablation from 2 d.p.f. when RGC axons arrive at the tectum led to formation of erroneous axon branches reaching outside the tectal neuropil as well as enlarged sizes of individual RGC arbors (Fig. 2e,f,h,i). To exclude that this phenotype was mediated indirectly by microglia clearing dying OPCs or by diverting microglial activities, which have an established role at eliminating synapses^16^, we carried out two controls: (1) genetic depletion of OPCs without causing inflammation by morpholino injection against oligodendrocyte transcription factor 2 (*olig2*) and (2) depletion of microglia using a morpholino against interferon regulatory factor 8 (*irf8*) (Fig. 2d and Extended Data Fig. 3b–d). Although *olig2* morphants also exhibited ectopic branching and enlarged RGC arbors, none of these phenotypes was seen in *irf8* morphants (Fig. 2g,j and Extended Data Fig. 3e,f). Therefore, erroneous RGC arborizations and enlarged RGC arbors resulted directly from the absence of OPCs.

**Fig. 3:**
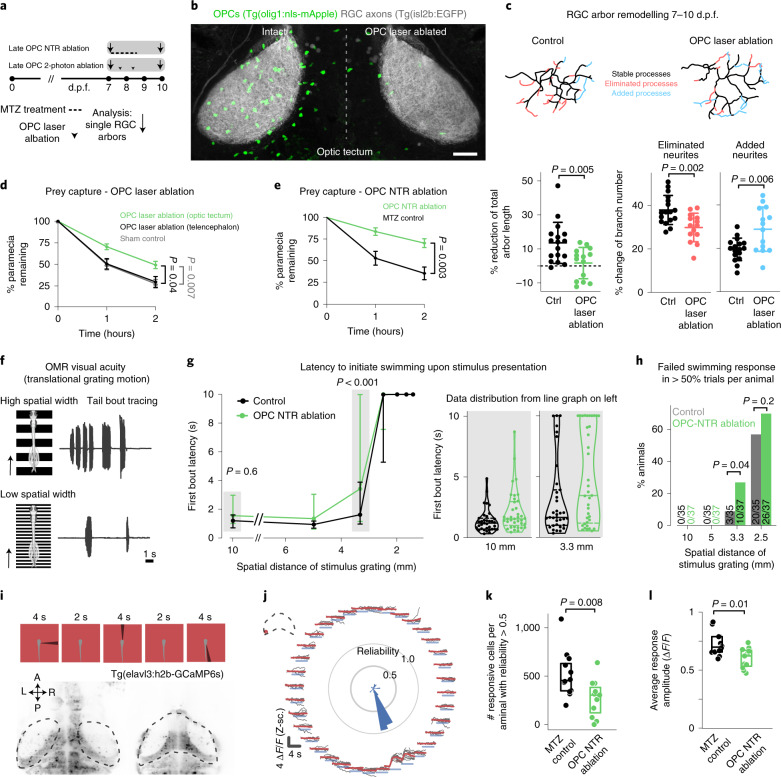
Late OPC ablation impairs RGC arbor remodeling and circuit function. **a**, Timelines of manipulations for late OPC ablations. **b**, Examples showing unilateral laser ablation of OPCs in the tectum. Scale bar, 20 µm. **c**, Reconstructions of time-projected RGC arbors highlighting stable, eliminated and added processes. Quantifications show diminished developmental reduction of RGC arbors between 7 d.p.f. and 10 d.p.f in OPC-ablated animals (left graph), mediated by decreased branch eliminations (middle) and enhanced additions (right) (left: mean 13.6 ± 12.1 s.d. in control versus 1.7 ± 9.2 in OPC laser ablation; middle: 37.9 ± 6.7 versus 29.9 ± 6.6; right: 19.5 ± 5.2 versus 28.8 ± 10.2; *n* = 17/14 cells in 11/9 animals from four experiments, unpaired two-tailed *t*-test, left: *t* = 3.022, d.f. = 29; middle: *t* = 3.372, d.f. = 29; right: *t* = 3.089, d.f. = 18.44). **d**, **e**, Impaired paramecium capture rates upon tectal OPC laser ablation (**d**) and OPC NTR ablation (**e**); (**d**: mean 27.6 ± 4.9 s.e.m. in sham control versus 29.7 ± 6.3 in telencephalic OPC ablation versus 49.2 ± 4.2 in tectal OPC ablation at 2-h time point, *n* = 11/10/23 animals from four experiments, two-way ANOVA, *F*_4,123_ = 3.369); (**e**: mean 35.2 ± 7.3 s.e.m. in MTZ control versus 70.2 ± 5.1 in OPC NTR ablation at 2-h time point, *n* = 18 animals per group from four experiments, two-way ANOVA, *F*_2,102_ = 6.759). **f**, Experimental setup of OMR elicited by moving gratings of different spatial widths and example trace of tail bout recording. **g**, First bout latencies in OMR assays. Violin plots show distribution of individual data points at 10 mm and 3.3 mm spatial frequency (10 mm: median 1.2 ± 1.6/0.7 IQR in control versus 1.5 ± 2.9/1.0 in OPC NTR ablation; 3.3 mm: median 1.6 ± 3.9/0.9 in control versus 3.4 ± 9.9/1.1 in OPC NTR ablation; *n* = 35/37 animals from six experiments, two-way ANOVA, *F*_6,490_ = 1.853). **h**, Enhanced possibility of failure to initiate swimming in response to narrow moving gratings upon OPC NTR ablation (one-tailed Fisher’s exact test). **i**, Top, visual stimulation protocol for analyzing responses of tectal neurons. Bottom, example anatomies obtained from calcium imaging in two different planes. Dashed lines indicate optic tectum. **j**, Visual responses from an example neuron. Each plot reports individual (black) and average (red) responses; plot position indicates position of the stimulus (top is frontal). Polar histogram represents the reliability score for this neuron to each stimulus position. **k**, Decreased number of reliably responsive neurons in OPC-ablated animals (median 457 ± 351/633 IQR in control versus 307 ± 123/386 in OPC NTR ablation, two-tailed Mann–Whitney *U*-test, *U* = 2.6465, *n* = 12/11 animals from three experiments). **l**, Decreased response amplitudes in OPC-ablated animals (median 0.700 ± 0.658/0.791 IQR in control versus 0.624 ± 0.536/0.672 in OPC NTR ablation, two-tailed Mann–Whitney *U*-test, *U* = 2.4618, *n* = 12/11 animals from three experiments).

**Extended Data Fig. 4:**
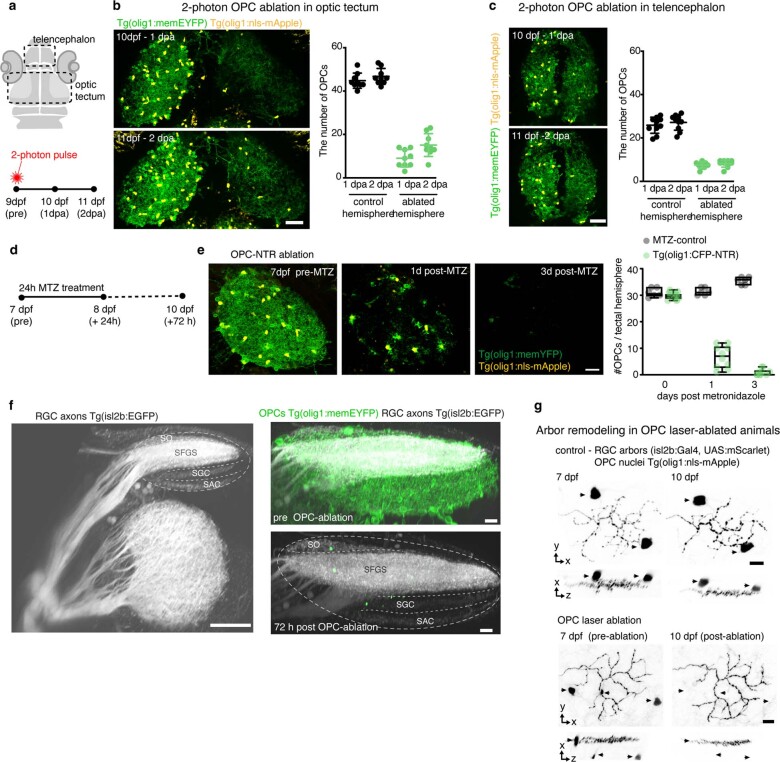
Characterization of late OPC ablation methods. **a)** Top: drawing of zebrafish head delineating the position of the optic tectum and telencephalon as shown in panels (b) and (c). Bottom: Schematic of OPC laser ablation paradigm. **b)** Dorsal views of OPC reporter lines showing the optical tectum after unilateral OPC ablation using 2 photon laser pulses and quantification of OPC numbers per tectal hemisphere. Data are expressed as mean ± S.D. (44.8 ± 3.5 in control *vs*. 9.0 ± 4.0 in ablated animals at 1 dpa; 46.8 ± 3.7 in control *vs*. 15.1 ± 5.2 in ablated animals at 2 dpa; n=9 animals per group from three experiments). Scale bar: 50 µm. **c)** Dorsal views of OPC reporter lines showing the telencephalon after unilateral OPC ablation using 2 photon laser pulses and quantification of OPC numbers per tectal hemisphere. Data are presented as mean ± S.D. (25.4 ± 3.7 in control *vs*. 7.1 ± 1.4 in ablated animals at 1 dpa; 26.7 ± 3.6 in control *vs*. 7.7 ± 1.6 in ablated animals at 2 dpa; n=10 animals per group from four experiments). Scale bar: 50 µm. **d)** Schematic of OPC NTR ablation paradigm in panels e and f. **e)** Example images with OPC NTR ablation and quantification of OPC numbers at different time points after MTZ treatment of Tg(olig1:CFP-NTR) animals. Boxes represent the median (center line) and I.Q.R. (32±30/33 cells in control *vs*. 30±29/36 cells in NTR animals before MTZ treatment; 32±30/34 cells in control *vs*. 8±4/10 cells in NTR animals at 1 day after MTZ treatment; 34±32/36 cells in control *vs*. 1±0/3 cells in NTR animals at 3 days after MTZ treatment), and whiskers indicate the minimum and maximum values. n=8/14 animals in MTZ-control/OPC NTR ablation from three experiments. **f)** Left: 3D rotation view of RGC axon projections showing major projection layers in the tectal neuropil: stratum opticum (SO), stratum fibrosum et griseum superficiale (SFGS), stratum griseum centrale (SGC), stratum album centrale (SAC). Representative images from 12 animals in five independent experiments. Scale bar: 50 µm. Right: Overall layering of RGC projections remains intact after OPC ablation. Representative images from 7 animals in three experiments. Scale bar: 10 µm. **g)** Confocal images of individual RGC axon arbors at 7 and 10 dpf in control and OPC ablated animals (n numbers as in Fig. 3c). Arrowheads depict OPCs. Scale bars: 10µm. See Fig. 3c for traces.

**Extended Data Fig. 5:**
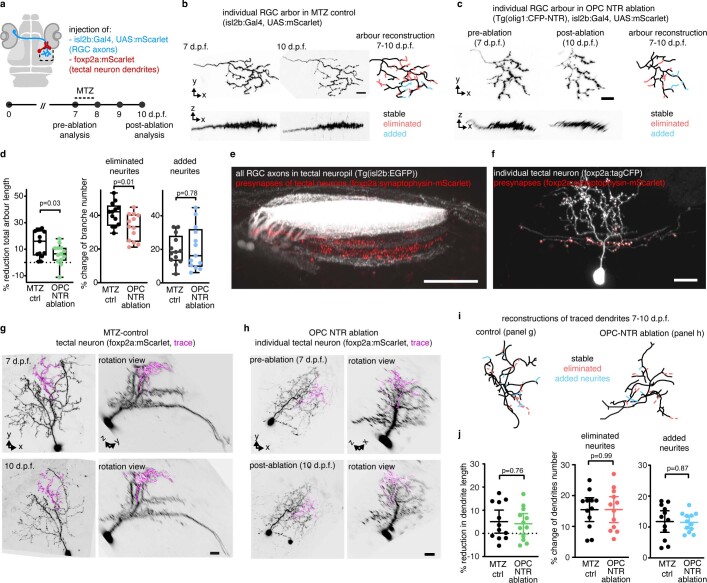
Remodeling of RGC axon arbors and periventricular interneuron (PVIN) dendrites after late OPC NTR ablation. **a)** Schematic of experimental paradigm used in this figure. **b-c)** Confocal images of individual RGC axon arbor in control and OPC NTR ablation animals. Tracing reveals stable, added, and eliminated processes (n numbers as in panel d). Scale bar: 10µm. **d)** Quantification showing RGC arbor length reduction, eliminated and added processes; Boxes represent the median (center line) and I.Q.R. (left:16.0 ± 5.5/24.1% in control *vs*. 6.6 ± 1.5/10.9% in OPC NTR ablation; middle: 42.1 ± 34.0/45.7% *vs*. 33.3 ± 24.4/40.4%; right: 18.4 ± 13.3/27.9% *vs*. 16.2 ± 9.8/31.9%), and whiskers indicate the minimum and maximum values; unpaired two-tailed t-test, left: t=2.278, d.f.=24; middle: t=2.652, d.f.=24; right: t=0.288, d.f.=24; n=13 cells per group in 12/10 animals in control/OPC NTR ablation from four experiments). **e)** Example image showing that pre-synapses of PVIN localize to deeper layers of tectal neuropil marked by Tg(isl2b:EGFP) labelling all RGC axons (two biological / one technical replicate). Scale Bar: 50µm. **f)** Example image of individual PVIN co-labelled using foxp2a:tagCFP to outline cell morphology and pre-synapse marker to reveal its axon (three biological / one technical replicate). Scale bar: 10 µm. **g**-**i)** Confocal images and rotation views of individual PVIN at two timepoints in control animals (g) and in OPC ablated animals (h) (n numbers as in panel j). Traces show dendritic processes analyzed in panel i. Scale bars: 10µm. **j)** Quantifications of dendritic PVIN arbor remodeling between 7-10dpf in control and OPC NTR ablation animals showing no significant changes in total dendritic length, eliminated and added processes number; n=12 cells in 12 animals in control and n=12 cells in 10 animals in OPC NTR from five experiments (left: mean 5.1±7.9 S.D. in control *vs*. 4.2 ± 7.0 in OPC NTR ablation; middle: 15.4 ± 6.0 *vs*. 15.5±6.6; right: 11.8±5.5 *vs*. 11.5 ± 3.1; unpaired two-tailed t test, left: t=0.305, d.f.=21.68; middle: t=0.007, d.f.=21.84; right: t=0.157, d.f.=17.52).

After their formation, RGC arbors undergo a phase of developmental pruning during larval stages when retinotectal connectivity is refined^17,18^. To test whether OPCs also play a long-lasting role during the refinement of RGC arbors as they continue to persist and interact with each other, we carried out late OPC ablations starting from 7 d.p.f. when zebrafish have a functional visual system. Ablations were carried out analogously using NTR-mediated chemogenetics or by two-photon-mediated cell ablation to specifically eliminate OPCs from the tectum (Fig. 3 and Extended Data Fig. 4). In control animals, individual RGC arbors underwent process remodeling with additions and eliminations of multiple neurites that lead to a net reduction in arbor size by about 14% between 7 d.p.f. and 10 d.p.f., similarly to previous reports (Fig. 3c and Extended Data Figs. 4g and 5a–d)^18^. This reduction in arbor size was significantly decreased in OPC-ablated animals (1.7% after OPC laser ablation and 6% after OPC NTR ablation), with some arbors even increasing in size due to a reduction in neurite eliminations and an increase in neurite additions after laser-mediated OPC ablation (Fig. 3c and Extended Data Figs. 4g and 5a–d). Despite changes in size, individual arbors remained stratified (Extended Data Figs. 4g and 5b,c). Furthermore, the effects on neurite remodeling were specific to axonal processes because remodeling of tectal neuron dendrites in the same tissue was unaffected upon OPC ablation, further corroborating that the effects observed did not result from unspecific collateral damage induced by our manipulations (Extended Data Fig. 5e–j).

**Extended Data Fig. 6:**
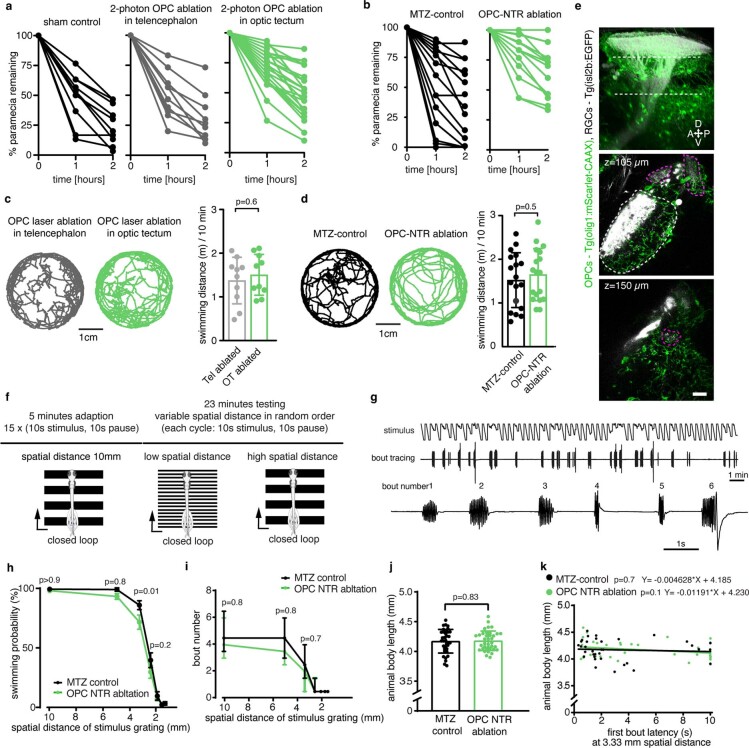
Analysis of zebrafish larvae after late OPC ablations. **a** and **b)** Quantification of paramaecium capture rates after OPC laser ablation (a) and OPC NTR ablation (b) showing measurements of individual animals over time. (a) n=11/10/23 animals from four experiments; (b) n=18 animals per group from four experiments. See also Fig. 3d and e. **c)** Example traces of the distance covered by individual freely swimming zebrafish larva within 10 minutes in control and OPC laser ablated animals. Right: quantification showing no significant change of freely swimming distance. (mean 1.37±0.53 S.D. in control vs. 1.51±0.47 in OPC laser ablation, unpaired two-tailed t test, t=0.586, d.f.=18, n=10 animals per group from four experiments). **d)** Example traces of the distance covered by individual freely swimming zebrafish larva within 10 minutes in control and OPC NTR ablated animals. Right: quantification showing no significant change of freely swimming distance. (mean 1.52±0.63 S.D. in control vs. 1.65±0.60 in NTR-OPC ablation, unpaired two-tailed t test, t=0.622, d.f.=34, n=18 animals per group from three experiments). **e)** Rotation view (top) and single planes (middle and bottom) of confocal images showing that OPCs also surround RGC arbors in pretectal regions. Dashed lines in top image indicate position of single plane images. Dashed white outline in single plane image demarcates tectal neuropil whereas magenta outlines indicate pretectal areas. Representative images from 3 animals in one experiment. Scale bar: 20 µm. **f)** Schematic of closed-loop OMR assay used. **g)** Representative tracing of an individual animal revealing different swim bouts to closed-loop stimuli in panel f. **h)** Reduced swimming probability in OPC NTR ablated animals with decreasing spatial distance of OMR stimulus grating; n=35/37 animals in control/OPC NTR from six experiments (mean with S.E.M., two-way ANOVA, F(6,490)=1.476). **i)** No significant changes observed in swim bout number exerted in either condition; n=35/37 animals in control/OPC NTR from six experiments. Data are expressed as median±I.Q.R.(4±3/6 in control *vs*. 3.5±2.5/5.5 OPC NTR ablation at 10mm; 4±2.5/5.5 in control *vs*. 3.5±2/5.5 OPC NTR ablation at 5mm; 2±1/3.5 in control *vs*. 1.5±0/3.5 OPC NTR ablation at 3.3mm; two-way ANOVA, F(6,490)=0.354). **j)** Quantification of animal body length between control and OPC NTR animals by 3 days post MTZ treatment shows no difference between treatment groups (mean 4.17±0.20 S.D. in control vs. 4.18±0.16 in OPC ablation, n=35/37 animals in control/OPC NTR from six experiments, unpaired two-tailed t test, t=0.184, d.f.=70). **k)** Quantification showing no correlation delay in first bout latency and animal body length. n=35/37 animals in control/OPC NTR from six experiments (two-tailed simple linear regression test, slope=−0.005/−0.012 in control and OPC NTR animals).

**Extended Data Fig. 7:**
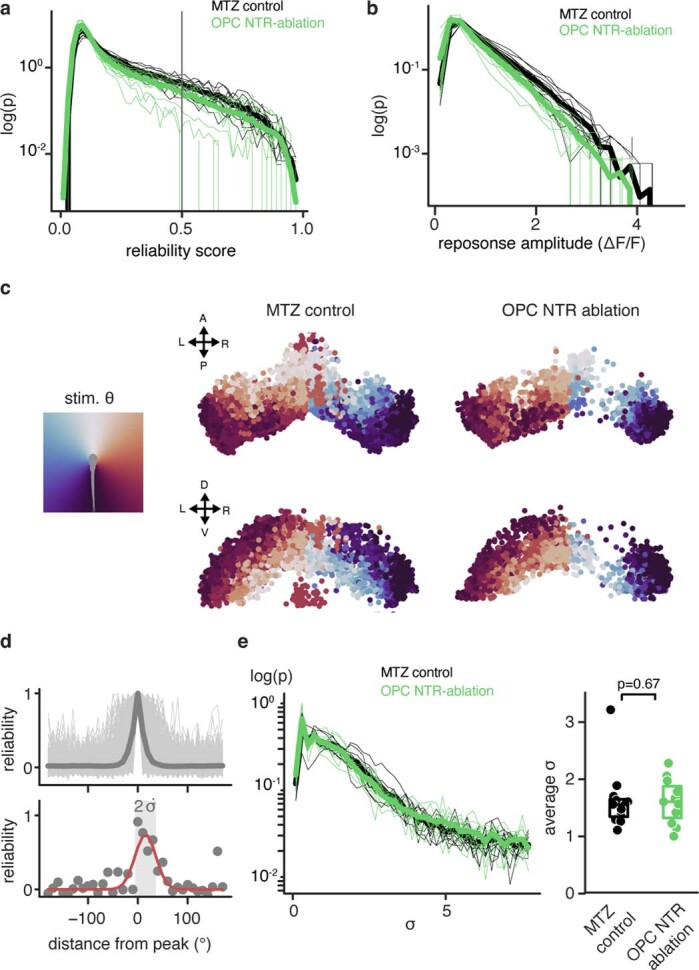
Functional Responses of PVIN neurons. **a)** Histogram of the maximum reliability score in all ROI (neurons) of each animal (thin lines) and group average (thick lines). The vertical line represent threshold for ROI to be considered visually responsive. **b)** Histogram of the average absolute response amplitude of all ROI (neurons) in each animal (thin lines) and group average (thick lines). **c)** Scatter plot representing location of all responsive cells in the tectum, color-coded by the position of the stimulus to which they responded most reliably. Although overall number of responsive cells is reduced in the ablation group, the retinotopy of the responses is preserved. **d)** Top: reliability scores of all responsive neurons for all stimuli, centered on the position of maximum responsiveness for each neuron and normalized to its maximum reliability. Dark gray line represents the average, light grey areas represent individual data points. Bottom: a gaussian line (red) was fit to each neuron’s responses (gray dots, data from an example neuron). The variance of the distribution was taken as a metric of the receptive field size of the neuron. The bar marks the 2 sigma interval from the sigmoidal fit of the reliability profile of an example cell. **e)** Histogram of the response curve variance of all ROI in each fish (thin lines) and group average (thick lines). (right) The average receptive field size was not significantly altered in the OPC ablated fish. Boxes represent median (center line) and I.Q.R. (1.541±1.329/1.633 in control *vs*. 1.590±1.306/1.861 in OPC-NTR ablation), the scatter dots represent the data for individual fish; two-tailed Mann–Whitney U test, U=−0.4308, n=12 MTZ-control animals and n=11 OPC-ablated animals from three experiments).

It has been reported that enlarged RGC arbors impair visual processing^19^. To test if OPC ablation affects functional performance as the visual system matures, we first carried out prey capture assays in late ablations from 7 d.p.f. onwards. These experiments showed that about two times more paramecia remained uncaptured in OPC NTR-ablated animals, and about 70% more paramecia remained uncaptured after unilateral laser ablation of tectal OPCs (Fig. 3d,e and Extended Data Fig. 6a,b). This effect was specific to tectal OPCs as OPC laser ablation from telencephalic regions did not impair paramecium capture (Fig. 3d and Extended Data Fig. 6a). None of our manipulations affected overall swimming activity, ruling out that gross locomotor defects account for reduced hunting (Extended Data Fig. 6c,d). Furthermore, we performed optomotor response (OMR) assays stimulated by moving gratings of different widths (Extended Data Fig. 6f,g and Supplementary Video 8). Narrower gratings become increasingly difficult to resolve, leading to longer latencies and ultimate failure to elicit OMR (Fig. 3f). After global NTR-mediated OPC ablation, animals were able to robustly elicit OMR in response to wide gratings (10-mm width), but there was a significantly increased probability of failure to initiate OMR when narrow gratings (3.3 mm) were presented (Fig. 3g,h and Extended Data Fig. 6h–k). It is known that OMR does not primarily require the tectum but, rather, pre-tectal areas to where RGC axons can extend collaterals in addition to the tectum^20,21^ and in which OPCs also reside (Extended Data Fig. 6e). Therefore, to test if OPCs within the tectum are of direct importance for functional sensory integration, we carried out in vivo calcium imaging of tectal neurons using Tg(elavl3:h2b-GCaMP6s) in response to light flashes in random positions around the visual field (Fig. 3i,j). Visual responses in OPC NTR-ablated animals were less reliable (Fig. 3k) and smaller in amplitude (Fig. 3l), whereas receptive field size and overall retinotopy along the tectum remained intact (Extended Data Fig. 7). Together, these data show that OPC ablation impairs visual processing.

In summary, our data reveal a physiological role for OPCs in fine-tuning the structure and function of neural circuits that is independent of their traditional role in myelin formation and which is mediated by regulating growth and remodeling of axon arbors. It remains an open question if regulation of arbor growth and remodeling by OPCs at different developmental stages might be mediated by different mechanisms, as OPCs constantly express growth-inhibitory molecules such as chondroitin sulfate proteoglycans^22^, but have also been reported to phagocytose axons^23^, similarly to how microglia can prune axons^24^. Do OPCs guide or prune axons, or both? Independent of the glial mechanism, how do changes in axon arbors translate into impaired neuronal connectivity underlying visual processing? Future work will reveal these mechanisms.

## Methods

### Zebrafish lines and husbandry

We used the following existing zebrafish lines and strains: Tg(mbp:nls-EGFP)^zf3078tg^^25^, Tg(mbp:memRFP)^tum101tg^^26^, Tg(mbp:memCerulean)^tum102tg^^26^, Tg(olig1:memEYFP)^tum107tg^^10^, Tg(olig1:nls-mApple)^tum109tg^^10^, Tg(olig1:mScarlet-CAAX)^27^ Tg(olig1:nls-Cerulean)^tum108tg^^10^, Tg(mfap4:memCerulean)^tum104tg^^10^, Tg(isl2b:EGFP)^zc7tg^^28^, Tg(elavl3:h2b-GCaMP6)^jf5tg^^29^, AB and nacre. The transgenic line Tg(olig1:CFP-NTR) was newly generated for this study. All animals were kept at 28.5 °C with a 14-h/10-h light/dark cycle according to local animal welfare regulations. All experiments carried out with zebrafish at protected stages were approved by the government of Upper Bavaria (Regierung Oberbayern, Sachgebiet 54; ROB-55.2-1-54-2532.Vet_02-18-153, ROB-55.2-2532.Vet_02-15-199 and ROB-55.2-2532.Vet_02-15-200 to T.C.) and the Animals in Science Regulation Unit of the UK Home Office (PP5258250 to David Lyons).

### Transgenesis constructs

To generate the middle entry clone pME_EYFP, the coding sequence was PCR amplified from a template plasmid using the primers attB1_YFP_F (GGGGACAAGTTTGTACAAAAAAGCAGGCTGCCACCATGCTGTGCTGC) and attB2R_YFP_R (GGGGACCACTTTGTACAAGAAAGCTGGGTCTTACTTGTACAGCTCGTCCATGC). The PCR product was recombination cloned into pDONR221 using BP clonase (Invitrogen).

The expression constructs pTol2_olig1:mScarlet, pTol2_olig1:EYFP, pTol2_olig1:tagCFP, pTol2_olig1:tagCFP-NTR, pTol2_cntn1b:mScarlet, pTol2_foxp2A:mScarlet and pTol2_foxp2A:Synaptophysin-mScarlet were generated in multi-site LR recombination reactions with the entry clone described above, p5E_olig1 (ref. ^26^), p5E_cntn1b^30^, p5E_foxp2A (gift from Martin Meyer, King’s College London)^31^, pME_tagCFP^26^, pME_mScarlet^10^, pME_Synaptophysin-nostop^10^, p3E_NTR-pA^25^, p3E_pA and pDestTol2_pA of the Tol2Kit^32^. The expression construct pTol2_isl2b:Gal4 using a published isl2b promoter clone^33^ was a kind gift of Leanne Godinho (TU Munich) and originally provided by Rachel Wong (Washington University); pTol2_olig1:memEYFP and pTol2_10xUAS:mScarlet were published previously^10^.

### DNA microinjection for sparse labeling and generation of transgenic lines

Fertilized eggs at one-cell stage were microinjected with 1 nl of a solution containing 5–20 ng μl^−1^ of DNA plasmid and 20 ng μl^−1^ of Tol2 transposase mRNA. Injected F_0_ animals were either used for single-cell analysis or raised to adulthood to generate full transgenic lines. For this, adult F_0_ animals were outcrossed with wild-type zebrafish, and F_1_ offspring were screened for presence of the reporter transgene under a fluorescence stereo dissecting microscope (Nikon SMZ18). ‘Tg(promoter:reporter)’ denotes a stable transgenic line, whereas ‘promoter:reporter’ alone indicates that a respective plasmid DNA was injected for sparse labeling of individual cells.

### OPC ablation using NTR

For NTR-mediated OPC ablation at early developmental stages, Tg(olig1:CFP-NTR) zebrafish at 2 d.p.f. were incubated in 10 mM metronidazole (MTZ) dissolved with 0.2% DMSO in 0.3× Danieau’s solution for 48 h at 28 °C in the dark, with a change of solution after 24 h. After MTZ incubation, embryos were rinsed and kept in 0.3× Danieau’s solution until analysis.

For NTR-mediated OPC ablation at later larval stages, Tg(olig1:CFP-NTR) zebrafish at 7 d.p.f. were incubated in 10 mM MTZ dissolved with 0.2% DMSO in 0.3× Danieau’s solution for 24 h at 28 °C in the dark. After MTZ incubation, larvae were rinsed and kept in nursery tanks with standard diet until 10 d.p.f. Non-NTR-expressing zebrafish treated with 10 mM MTZ were used as controls in all experiments.

### OPC ablation using two-photon lasers

OPCs were laser ablated from Tg(olig1:nls-mApple) using an Olympus FV1000/MPE equipped with a MaiTai DeepSee HP (Newport/Spectra Physics) and a ×25 1.05 NA MP (XLPLN25XWMP) water immersion objective. Continuous confocal scans using a 559-nm laser were taken to locate individual OPC nuclei in the optic tectum, which was identified by additional transgenic genetic markers (Tg(olig1:memYFP) or Tg(isl2b:EGFP)). Each cell was ablated using a 500-ms line scan across the cell nucleus using the MaiTai laser tuned to 770 nm (1.75-W output). The wavelength was incrementally increased for ablating OPCs in deeper tissue. After successful ablation, previously bright, round nuclei appeared dim, irregular or fragmented. The ablation procedure was repeated when cells did not show this signature. Unilateral OPC ablations took 60–90 min in the tectum. For analysis of axon remodeling after OPC laser ablation, surviving and/or repopulating OPCs were ablated again on the second day.

### Morpholino-mediated depletion of microglia and OPCs

Microglia were depleted from zebrafish embryos by microinjection of 4.5 pg of a previously published morpholino targeting the start codon of *irf8* (5′-TCAGTCTGCGACCGCCCGAGTTCAT-3′)^34^. OPCs were depleted by microinjection of 7.5 pg of a previously published morpholino targeting the start codon of *olig2* (5′-ACACTCGGCTCGTGTCAGAGTCCAT-3′)^35^. Both morpholinos were synthesized by Gene Tools.

### Neutral red staining

Zebrafish embryos were incubated for 2.5 h in the dark in 2.5 µg ml^−1^ of neutral red solution (Sigma-Aldrich, n2889) diluted in Danieau’s solution. Afterwards, embryos were washed three times for 10 min with Danieau’s solution. Bright-field images of the head of the fish were taken using a Leica DFC300 FX Digital Color Camera.

### Prey capture assay

Next, 2 ml of 0.3× Danieau’s solution with 30 *Paramecium multimicronucleatum* were added to a 35-mm dish, along with a single zebrafish larva. The number of remaining paramecia was determined at hourly intervals for 2 h. To rule out batch-dependent effects resulting from ‘natural’ paramecia death followed by their disintegration, a control containing paramecia but no fish was run alongside each experiment. Spontaneous paramecia death occurred only rarely in 0–3%.

### Locomotor activity assay

For all experiments, testing occurred between 9:00 and 17:00 using a randomized trial design to eliminate systematic effects due to the time of day. A tracking chamber was prepared by using a 35-mm Petri dish mold surrounded by 1% agarose situated in the center of a 85-mm Petri plate to eliminate mirroring that occurs at the wall of a plastic Petri dish. Single zebrafish were placed into the well filled with 2 ml of 0.3× Danieau’s solution. The plate was positioned above an LED light stage to maximize contrast for facilitating zebrafish tracking (two dark eyes and swim bladder of zebrafish larva on a light background), with a high-speed camera (XIMEA MQ013MG-ON) equipped with a Kowa LM35JC10M objective positioned above the dish. The larvae acclimated to the recording arena for 5 min before the start of video acquisition. The center of mass of two eyes and swim bladder was taken as the center of the fish. Subsequently, video of spontaneous free swimming was recorded for 10 min at 100 Hz using a custom-written Python script and the Stytra package^36^.

### Optomotor response assay

Zebrafish larvae were embedded in 1% agarose in a 35-mm Petri dish. After allowing the agarose to set, the dish was filled with 0.3× Danieau’s solution, and the agarose around the tail was removed with a scalpel, leaving the tail of fish free to move (hereby referred to as a head-restrained preparation)^37^. Visual stimuli were presented on the screen from below using an ASUS P3E micro projector and an infrared light (Osram 850-nm high-power LED). The fish’s tail was tracked using a high-speed camera (XIMEA MQ013MG-ON) and a 50-mm telecentric objective (Navitar TC-5028). A square-wave grating with variable spatial period and maximal contrast was achieved by the projector (black and white bars), and online tail tracking and stimulus control was performed using Stytra software 0.8.26 (ref. ^36^). Experiments were performed in closed loop, meaning that the behavior of fish was fed back to the visual stimulus to provide the fish with visual feedback. Therefore, when the fish swam, the grating accelerated backward at a rate proportional to swim power—that is, [stimulus velocity] = 10 – [gain] × [swim power]. Swim power was defined as the standard deviation of the tail oscillation in a rolling window of 50 ms. To obtain a feedback that mimics the visual feedback that the animal would receive when freely swimming, the gain multiplication factor was chosen to result in an average fictive velocity of about 25 mm s^−1^ during the bout. When the fish was not swimming, [swim power] = 0, the grating moved in a caudal to rostral (forward) direction at a baseline speed of 10 mm s^−1^. The stimulus scene was a square window that was centered on the head of fish and spanned a field of total 60 × 60 mm. For analysis, individual bouts were counted as episodes where the swim power was above 0.1 radian for at least 100 ms. Then, latency to first bout and total number of bouts were quantified for each trial (latency was set as a default value equal to the stimulus duration, when fish did not respond). Analysis was performed with custom scripts written in Python.

### Zebrafish mounting for live-cell microscopy

Zebrafish larvae were anaesthetized with 0.2 mg ml^−1^ of 3-aminobenzoic acid ethyl ester (MS-222). For confocal microscopy, animals were mounted ventral side up in 1% ultra-pure low-melting-point agarose (Invitrogen) onto a glass-bottom 3-cm Petri dish (MatTek). For two-photon microscopy, embryos were mounted ventral side up in low-melting-point agarose on a glass coverslip. The coverslip was then flipped over on a glass slide with a ring of high-vacuum grease filled with a drop of 0.2 mg ml^−1^ of MS-222 to prevent drying out of the agarose. After imaging, the animals were either euthanized or released from the agarose using microsurgery forceps and kept individually until further use.

### Immunohistochemistry

Samples were fixed overnight at 4 °C in a solution of 4% paraformaldehyde in PBS solution containing 1% Tween 20. After fixation, the samples were washed in the same solution without fixative and blocked for 1.5 h at room temperature in PBS buffer, 0.1% Tween 20, 10% FCS, 0.1% BSA and 3% normal goat serum. Primary antibody incubation was conducted at 4 °C overnight in blocking solution. Afterwards, samples were washed three times in PBS with 0.1% Tween 20 and then incubated with Alexa Fluor 633-conjugated secondary antibody. Stained samples were washed three times in PBS with 0.1% Tween 20 and subsequently mounted with ProLong Diamond Antifade Mountant (Thermo Fisher Scientific). Primary antibody rabbit anti-HuC/D (Abcam, ab210554) was used at a dilution of 1:100. Goat anti-rabbit secondary antibody (Thermo Fisher Scientific) was used at a dilution of 1:1,000. Images were obtained using a confocal microscope (Leica TCS SP8).

### Confocal microscopy

Images of embedded zebrafish were taken with a Leica TCS SP8 confocal laser scanning microscope (LASX 3.5.2.18963). We used 458 nm for excitation of Cerulean and tagCFP; 458 and 488 nm for EGFP; 514 nm for EYFP; 561 nm for mApple and mScarlet; and 633 nm for AF633 and BODIPY630/650. For overview images and analysis of cell numbers based on nuclear transgenes, we used a ×10 / 0.4 NA objective (acquisition with 568-nm pixel size (*x*–*y*) and 2-μm *z*-spacing). For all other analyses, we acquired 8-bit or 12-bit confocal images using a ×25 / 0.95 NA water objective with 114–151-nm pixel size (*x*–*y*) and 1-μm or 1.5-μm *z*-spacing.

### Analysis of contact-mediated retraction between RGC axons and OPC processes

For analyzing dynamic interactions between RGC axon arbors and OPC processes, images were taken over 2 h within 2-min intervals and 1-μm z-spacing. Three-dimensional (3D) movies were subsequently generated for analyzing RGC retraction using Imaris software. Contact-mediated repulsion was classified as every event in which the tip of an extending RGC process directly opposed or apposed an OPC process, followed by RGC process retraction to resolve this apposition within the following seven frames. RGC processes, which changed from extension to retraction without such prior contact to OPC processes, were categorized as contact-independent retraction.

### Analysis of axonal and dendritic arbor remodeling

Only RGC axons that arborized within the tectal neuropil and that could be traced back to the optic nerve were included for analysis. The neurites of periventricular interneurons (PVINs) extending to the superficial layers were randomly selected for analysis, as the dendrites of PVINs located in the superficial layers and their axons are located in deeper layers^38^. Individual axonal and dendritic arbors were analyzed using the segmentation tool of 3D tracing in the simple neurite tracer plugin in Fiji/ImageJ^39^. Each arbor was traced from its first branch point out to all branch tips, and each branch segment was counted from the branch point to the next branch point or branch tips. From this tracing, we extracted the measurement of total branch length and the total number of branch segments. The eliminated/added branch segment was obtained by comparing the tracing of the same neuron at two time points.

### Lightsheet imaging for functional calcium imaging of tectal neurons

For lightsheet imaging, MTZ-treated Tg(olig1:CFP-NTR), Tg(elavl3:h2b-GCaMP6s) with late OPC ablation and MTZ-treated control Tg(elavl3:h2b-GCaMP6s) fish were embedded at the center of a custom-built plastic chamber using 2–2.5% low-melting-point agarose. The chamber was then placed onto the stage of a custom-built lightsheet microscope, previously described in ref. ^40^. In brief, light from a 473-nm laser (Cobolt) was scanned with galvanometric mirrors (Sigmann Electronik) horizontally to create a ~5-µm-thick excitation sheet. The sheet was then moved vertically together with the light collection objective, controlled with a piezo controller (Piezosystem Jena). The eyes were protected from the incoming light by two plastic screens positioned at the conjugate plane of the scanning laser focus. Two orthogonal sheets were generated—one impinged on the brain from the side, the other one from the front—to ensure extensive coverage of the whole brain without hitting the eyes. The image was filtered with a band-pass 525/50 filter and acquired using an ORCA-Flash version 4.0 camera (Hamamatsu Photonics). The microscope was controlled using the Sashimi package (https://zenodo.org/record/4122062#.YQl7yC0RpQI). We acquired volumes of ~130 × 400 × 340 µm (dorso–ventral, left–right and anterior–posterior axes, respectively) at a resolution of 15 × 0.6 ×0.6 µm and a frame rate of 3 Hz.

Visual stimuli were projected on a white plastic screen placed below the fish. The protocol consisted of a sequence of dark flashes on a red background, spanning 10° circular sectors of the area around the fish for a total of 36 different locations. Flashes were shown for 4 s, with a pause of 2 s of flat red background between them, and presented ten times each in a sequence randomized differently for every fish. The script for generating the experimental protocol in Stytra is available in the code repository. Synchronization between the imaging and the stimulus presentation was achieved by ZMQ-mediated triggering between Stytra and Sashimi.

### Imaging data pre-processing for calcium imaging

Raw stacks from the lightsheet imaging were inspected, and fish with excessive drift were discarded, blindly to experimental group. Two of 25 fish were excluded, and all the remaining fish (*n* = 12 MTZ control fish and *n* = 11 OPC-ablated fish) were included for all subsequent analyses. The data were fed into suite2p for alignment and region of interest (ROI) segmentation. suite2p parameters were kept mostly at their standard values, adjusting values for cell side and temporal sampling frequency. The cell classification and the deconvolution steps were skipped in the suite2p pipeline, and Z*-*scored raw fluorescence extracted from all detected ROIs was used in all subsequent analyses. The script used for running the data pre-processing with suite2p is available in the code repository.

After alignment, a mask delineating the optic tectum was drawn manually for each fish using pipra^41^, to include only the ROI in this region for further analysis. However, responsiveness of all the best-scoring ROIs for this stimulus were located in the optic tectum, and the conclusions hold regardless of this selection criterion and the exact boundaries of the masks.

### Analysis of calcium responses to visual stimulus

To quantify responses of neurons to individual stimuli, the activity from individual ROIs was de-trended (subtracting the difference between the first and last points), Z-scored and chunked in a window of −2 s to 5 s from the stimulus onset. To compute the reliability score, we obtained the cross-correlation matrix across all stimuli repetitions and averaged all its off-diagonal values. For the average amplitude, the absolute value of the difference was calculated between the integral of the response in the 4 s of the stimulus and during the 2 s of the pre-stimulus pause. For the estimation of the receptive field size, ‘visually responsive’ (reliability score >0.5) ROIs were selected, and a Gaussian curve to the array of reliability scores was fit over stimulus positions. The variance of the Gaussian was taken as a measure of the width of the tuning curve. All statistical comparisons were performed using the Mann–Whitney *U*-test (with the implementation in scipy.stats.ranksums^42^).

### Image and data presentation

Images were analyzed with Fiji and Imaris. Morphology reconstructions were carried out with the Imaris FilamentTracer module. Data were prepared and assembled using GraphPad Prism 7, 8 and 9, Fiji and Adobe Illustrator CS6 and 2021.

### Statistics and reproducibility

For analyses that involved cohorts of animals or treatment groups, zebrafish embryos of all conditions were derived from the same clutch and selected at random before treatment. No additional randomization was used during data collection. For time-course analyses of OPCs and RGC, zebrafish were screened for single-cell labeling before imaging, and all animals with appropriate expression were used in the experiment. Two fish with excessive drift were discarded for calcium imaging, blindly to experimental group; no other data were excluded from the analyses. We selected sample sizes based on similar sample sizes that were previously reported^18,37,43,44^. No statistical analysis was used to pre-determine sample sizes. Data collection and analysis were not performed blinded to the conditions of the experiments unless stated. Analysis was performed using Microsoft Excel and GraphPad Prism. All data were tested for normal distribution using the Shapiro–Wilk normality test before statistical testing. In the figures, bar graphs are shown as mean ± s.d.; line plots are shown as mean ± s.e.m. or median ± interquartile range (IQR); box and whisker plots are expressed as median ± IQR, minimum and maximum values; and violin plots represent the median ± IQR, minimum and maximum values. For statistical tests of normally distributed data that compared two groups, we used unpaired *t*-tests. Non-normally distributed data were tested for statistical significance using the Mann–Whitney *U*-test (unpaired data). For multiple comparisons test, one-way ANOVA was used for parametric data and the Kruskal–Wallis test for non-parametric data (both with Benjamini–Krieger and Yekutieli correction). Repeated measurements were tested using two-way ANOVA with Sidak’s correction. We used Fisher’s exact test to analyze contingency tables.

### Reporting Summary

Further information on research design is available in the Nature Research Reporting Summary linked to this article.

## Online content

Any methods, additional references, Nature Research reporting summaries, source data, extended data, supplementary information, acknowledgements, peer review information; details of author contributions and competing interests; and statements of data and code availability are available at 10.1038/s41593-022-01023-7.

## Supplementary information

Reporting Summary

Supplementary Video 1 Animated *z*-projection of double transgenic olig1:memEYFP, mbp:memRFP zebrafish at 5 d.p.f. shown in Supplementary Fig. 1a.

Supplementary Video 2 Animated *z*-projection of double transgenic isl2b:EGFP, mbp:memRFP zebrafish at 5 d.p.f. shown in Supplementary Fig. 1b.

Supplementary Video 3 Animated rotation showing confocal images and tracing of individual tectal OPCs as in Supplementary Fig. 1g.

Supplementary Video 4 Time lapse of single RGC axon arbor in transgenic animals labeling all OPCs and microglia, showing continuous dynamic interaction between axon and OPC processes.

Supplementary Video 5 Reconstructed time lapse of single RGC axon in transgenic animal labeling all OPCs, showing RGC axon retraction with and without contact of OPC processes as in Fig. 2a.

Supplementary Video 6 3D rotation of time point 08:18 in Fig. 2a and Supplementary Video 5.

Supplementary Video 7 Animated *z*-planes of single RGC axon in transgenic animal labeling all OPCs as in Supplementary Fig.2b,c.

Supplementary Video 8 Testing OMR in head-fixed larval zebrafish using Stytra software. Tail bouts are elicited by presentation of moving gratings. Tracking of initiated bouts are fed back to decrease the moving stimulus speed (closed loop).

## Data Availability

All data underlying this study will be made available upon reasonable request. Raw data for functional analysis have been deposited at 10.5281/zenodo.5894603.

## Code availability

The codes used for running and analyzing behavioral and imaging experiments are available at 10.5281/zenodo.5894770.
