# Supplementary material for: Oligodendrocyte precursor cells sculpt the visual system by regulating axonal remodeling
Source: Nat Neurosci. 2022 Mar 3;25(3):280–4. doi: 10.1038/s41593-022-01023-7 (PMC8904260; doi:10.1038/s41593-022-01023-7)
Supplement: Supplementary file 1 — Reporting Summary [file 41593_2022_1023_MOESM1_ESM.pdf]

Corresponding author(s): Tim Czopka

Last updated by author(s): Jan 21, 2022

Reporting Summary

Nature Research wishes to improve the reproducibility of the work that we publish. This form provides structure for consistency and transparency in reporting. For further information on Nature Research policies, see our Editorial Policies and the Editorial Policy Checklist.

Statistics

For all statistical analyses, confirm that the following items are present in the figure legend, table legend, main text, or Methods section.

n/a	Confirmed	
☐	☒	The exact sample size (n) for each experimental group/condition, given as a discrete number and unit of measurement
☐	☒	A statement on whether measurements were taken from distinct samples or whether the same sample was measured repeatedly
☐	☒	The statistical test(s) used AND whether they are one- or two-sided<br>*Only common tests should be described solely by name; describe more complex techniques in the Methods section.*
☒	☐	A description of all covariates tested
☐	☒	A description of any assumptions or corrections, such as tests of normality and adjustment for multiple comparisons
☐	☒	A full description of the statistical parameters including central tendency (e.g. means) or other basic estimates (e.g. regression coefficient) AND variation (e.g. standard deviation) or associated estimates of uncertainty (e.g. confidence intervals)
☐	☒	For null hypothesis testing, the test statistic (e.g. F, t, r) with confidence intervals, effect sizes, degrees of freedom and P value noted<br>*Give P values as exact values whenever suitable.*
☒	☐	For Bayesian analysis, information on the choice of priors and Markov chain Monte Carlo settings
☒	☐	For hierarchical and complex designs, identification of the appropriate level for tests and full reporting of outcomes
☒	☐	Estimates of effect sizes (e.g. Cohen's d, Pearson's r), indicating how they were calculated

Our web collection on statistics for biologists contains articles on many of the points above.

Software and code

Policy information about availability of computer code

Data collection	LASX 3.5.2.18963 (Leica), Stytra 0.8.26 (Stih et al., 2019; PMID: 30958870), Fluoview FV1000 - MPE (Olympus), custom build lightsheet microscope (Markov et al., doi:10.1101/2020.02.12.945956)
Data analysis	Fiji v1.52o, Imaris 8.4.2 (Bitplane), NeuronJ 1.4.3 (plugin for Fiji, open source), Microsoft Excel 2016 (Microsoft), Prism V7, V8, V9 (GraphPad), Photoshop 2021 (Adobe), Illustrator 2021 (Adobe), Python 3.7, custom written analysis codes (https://github.com/portugueslab/xiao_et_al)

For manuscripts utilizing custom algorithms or software that are central to the research but not yet described in published literature, software must be made available to editors and reviewers. We strongly encourage code deposition in a community repository (e.g. GitHub). See the Nature Research guidelines for submitting code & software for further information.

Data

Policy information about availability of data

All manuscripts must include a data availability statement. This statement should provide the following information, where applicable:
- Accession codes, unique identifiers, or web links for publicly available datasets
- A list of figures that have associated raw data
- A description of any restrictions on data availability

The data that support the findings of this study are available from the corresponding author upon reasonable request.

Field-specific reporting

Please select the one below that is the best fit for your research. If you are not sure, read the appropriate sections before making your selection.

☒ Life sciences ☐ Behavioural & social sciences ☐ Ecological, evolutionary & environmental sciences

For a reference copy of the document with all sections, see nature.com/documents/nr-reporting-summary-flat.pdf

Life sciences study design

All studies must disclose on these points even when the disclosure is negative.

Sample size	No sample-size calculations were performed. Sample size was determined to be adequate based on similar sample sizes that have previously reported using similar experiments reported in references 18,37,43,44 of this manuscript, as stated in the Methods.
Data exclusions	Two animals with excessive drift during recording of calcium imaging were discarded, blindly to experimental group. No other data were excluded from the analyses.
Replication	All experiments were repeated at least 3 times and results could be replicated each time.
Randomization	For analyses that involved cohorts of animals or treatment groups, zebrafish embryos of all conditions were derived from the same clutch and selected at random. For time-course analyses of OPCs and RGC, zebrafish were screened for single-cell labeling before imaging, and all animals with appropriate expression were used in the experiment.
Blinding	Investigators were not blinded during data collection and analysis. Descriptive imaging data that do not compare groups cannot be blinded. When treatment groups have been compared, internal cross-counting by group members who did not perform the experiment has been performed for all experiments at random.

Reporting for specific materials, systems and methods

We require information from authors about some types of materials, experimental systems and methods used in many studies. Here, indicate whether each material, system or method listed is relevant to your study. If you are not sure if a list item applies to your research, read the appropriate section before selecting a response.

Materials & experimental systems

n/a	Involved in the study	
☐	☒	Antibodies
☒	☐	Eukaryotic cell lines
☒	☐	Palaeontology and archaeology
☐	☒	Animals and other organisms
☒	☐	Human research participants
☒	☐	Clinical data
☒	☐	Dual use research of concern

Methods

n/a	Involved in the study	
☒	☐	ChIP-seq
☒	☐	Flow cytometry
☒	☐	MRI-based neuroimaging

Antibodies

Antibodies used	1) anti-HuC/D (Abcam), catalog number: ab210554, polyclonal, dilution: 1/100.<br>2) anti-rabbit Alexa Fluor 633 (Themo Fisher), catalog number: A-21071, polyclonal, lot number: 1889315, Dilution: 1/1000.
Validation	This antibody has been validated previously in zebrafish (Lee et al., 2020; PMID:32396062)

Animals and other organisms

Policy information about studies involving animals; ARRIVE guidelines recommended for reporting animal research

Laboratory animals	Species: Danio Rerio; wildtype strains: AB with mitfa mutation; transgenic lines in mixed backgrounds: Tg(olig1:CFP-NTR), Tg(mbp:nls-EGFP), Tg(mbp:memRFP), Tg(mbp:memCerulean), Tg(olig1:memEYFP), Tg(olig1:nls-mApple), Tg(olig1:mScarlet-CAAX), Tg(olig1:nls-Cerulean), Tg(mfap4:memCerulean), Tg(isl2b:EGFP), Tg(elavl3:h2b-GCaMP6)); Sex: n/a; Age: between 2dpf and 14 dpf
Wild animals	The study did not involve wild animals.
Field-collected samples	The study did not involve samples collected from the field.

Ethics oversight	All experiments carried out with zebrafish at protected stages have been approved by the government of Upper Bavaria (Regierung Oberbayern - Sachgebiet 54; ROB-55.2-1-54-2532.Vet_02-18-153, ROB-55.2-2532.Vet_02-15-199, and ROB-55.2-289 2532.Vet_02-15-200 to TC) and the Animals in Science Regulation Unit of the UK Home Office (PP5258250 to Prof. David Lyons).

Note that full information on the approval of the study protocol must also be provided in the manuscript.
